# *Ggtree*: A serialized data object for visualization of a phylogenetic tree and annotation data

**DOI:** 10.1002/imt2.56

**Published:** 2022-09-28

**Authors:** Shuangbin Xu, Lin Li, Xiao Luo, Meijun Chen, Wenli Tang, Li Zhan, Zehan Dai, Tommy T. Lam, Yi Guan, Guangchuang Yu

**Affiliations:** ^1^ Department of Bioinformatics, School of Basic Medical Sciences Southern Medical University Guangzhou China; ^2^ State Key Laboratory of Emerging Infectious Diseases, School of Public Health The University of Hong Kong Hong Kong SAR China; ^3^ Joint Institute of Virology (Shantou University–The University of Hong Kong) Shantou University Shantou China

**Keywords:** annotation data, data structure, *ggtree*, phylogenetic tree, visualization

## Abstract

While phylogenetic trees and associated data have been getting easier to generate, it has been difficult to reuse, combine, and synthesize the information they provided, because published trees are often only available as image files and associated data are often stored in incompatible formats. To increase the reproducibility and reusability of phylogenetic data, the *ggtree* object was designed for storing phylogenetic tree and associated data, as well as visualization directives. The *ggtree* object itself is a graphic object and can be rendered as a static image. More importantly, the input tree and associated data that are used in visualization can be extracted from the graphic object, making it an ideal data structure for publishing tree (image, tree, and data in one single object) and thus enhancing data reuse and analytical reproducibility, as well as facilitating integrative and comparative studies. The ggtree package is freely available at https://www.bioconductor.org/packages/ggtree.

## Highlights

- The phylogenetic tree and diverse accompanying data can be stored in a *ggtree* graph object, which improves the reproducibility and reusability of phylogenetic data.
- The phylogenetic tree and associated data can be extracted from the *ggtree* object, which can be reanalyzed and help various scientific disciplines synthesize their comparative studies and phylogenetic information.
- The *ggtree* graph object can be rendered as a static image, and the visualization directives that were previously saved in the object can be reused to display a different tree object in a manner akin to Microsoft Word Format Painter.

## Graphical abstract

The *ggtree* object is designed to store phylogenetic tree and associated data, and the object itself is a graphic object that can be rendered as an image file. This work will increase the reproducibility and reusability of phylogenetic data, as well as facilitate integrative and comparative studies.

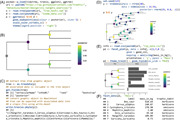

## INTRODUCTION

Phylogenetic data have enormous potential for reuse as phylogeny is becoming central to a wide range of research in ecology, evolutionary biology, epidemiology, and molecular biology. Reusing phylogenetic data can contribute to synthesizing phylogenetic knowledge and comparative analyses in a number of scientific disciplines. However, a previous survey alerts that ~60% of published phylogenetic data are lost to science forever [1]. One of the reasons for this situation is that phylogenetic trees are often published as static images and lack interoperable file formats for data sharing [2]. Creating tree figures annotated with associated data (taxonomy information, meta‐data, phenotypic and epidemiological data, etc.) is a routine practice. Although tools for tree visualization and annotation are proliferating, the dominant objective remains to produce a publication‐ready figure, which involves multiple steps in selecting the annotation data (e.g., bootstrap values) and rendering it on the tree (e.g., as text labels or branch colors). The process is one way and a dead end, yielding a static figure such that the underlying information cannot be reused. We need a paradigm shift from producing a static figure to a serialized data object that contains the tree, associated data, and visualization directives in addition to rendering it as a visualization graphic.

## RESULTS

### A graph object for managing and storing the phylogenetic tree and associated data

**Figure 1:**
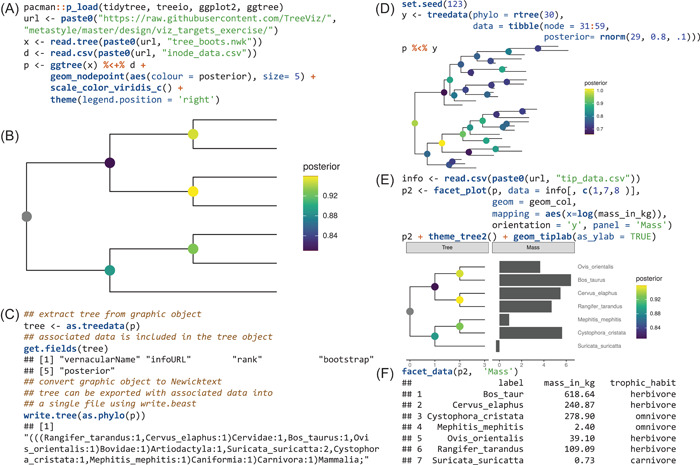
Examples of using *ggtree* object to store phylogenetic tree, associated data, and visualization directive. A tree in Newick format was read into R as *x* and an associated data, *d*, that stores posterior of internal nodes were used to visualize circle points that were colored by the posterior values. The output graphic object, that is, the *ggtree* object, was stored in *p* (A). The *ggtree* object, *p*, can be rendered as a picture by *print(p)* (B). The *ggtree* object contains the information of the phylogenetic tree and associated data. It can be converted back to a *treedata* object with the tree and all the associated data, including posterior, bootstrap, and other values in *d* that were attached to the graphic object, *p*. The tree can be exported to Newick text (without associated data) and BEAST Nexus file (with associated data) (C). The *ggtree* object contains visualization directives that can be reused to visualize a new tree object by applying its visualization style (D). Associated data can be visualized with the tree side by side using *facet_plot* (E). The data used in *facet_plot* can also be extracted from the graphic object using *facet_data* function (F).

Here, we describe a data structure, the *ggtree* object, defined in the *ggtree* package. *Ggtree* is an R/Bioconductor package for the visualization and annotation of phylogenetic trees with diverse associated data [3]. We previously proposed two methods for mapping and visualizing associated data on the phylogeny [4]. Taxon‐associated data can be linked to the tree structure within the *ggtree* object by the *%<+%* operator and complex associated data can be visualized by a specific *geom* layer in a separate panel and aligned to the tree based on the tree structure using *facet_plot* or *geom_facet* function [4]. Data that are mapped and visualized using these two methods are preserved and can be extracted from the *ggtree* object. In Figure 1, associated data were attached to the tree using the *%<+%* operator, and the posterior values were used to color circle points on the tree (Figure 1A). The output *ggtree* object is a graphic object and can be rendered as a static figure (Figure 1B). The object preserves the information of the phylogenetic tree as well as associated data that was attached (Figure 1C). Users can convert this graphic object to a *phylo* (only tree structure without annotation data) or *treedata* (tree structure and annotation data) object. The tree object can be further processed using the *tidytree* or *treeio* packages and can be exported to Newick, Nexus, or BEAST Nexus, which enables associated data to be stored as annotated elements [5]. The *ggtree* graphic object also contains visualization directives and the visualization style that can be reused to visualize a new tree object (Figure 1D), which is similar to Microsoft Word Format Painter. Data that are used in *facet_plot* can be complex and heterogeneous, such as genetic information at the pan‐genome scale and species abundance distributions. Although the data are not directly mapped to the tree, it can also be extracted from the *ggtree* object. The body weight information was used to be visualized as barplot with the tree side by side (Figure 1E) and it can be extracted from the *ggtree* graphic object using *facet_data* function (Figure 1F).

## DISCUSSION AND CONCLUSION

In addition to the tree itself, all the pieces of information that are mapped and visualized on the phylogeny using the methods proposed in *ggtree* [4] are reusable, including data that are rendered as visual characteristics to display the tree and data that are used to produce a graph to align with the tree. With *ggtree*, phylogenetic data as well as the visual directives that are used to create the image in a publication are seamlessly stored in a single object. This makes the visualization more portable and transparent enabling high‐quality phylogenetic information to be shared and reused in different projects. It also offers great potential for remote collaborators to modify tree data presentation. If a tree was published using the *ggtree* graphic object (i.e., as a supplemental file or deposited to a data repository), others can download and read the file into R to reproduce the figure that is identical to the published one and can add or modify layers of tree annotation by reusing data in the object or from other sources. Users can obtain tree structure and associated data from the *ggtree* object for integrative and comparative analyses using a number of R packages. A paradigm shift from generating a static figure to producing a serialized data object like *ggtree* not only enhances analytic reproducibility but also assures data reusability and facilitates synthetic and comparative studies to identify and pursue new questions. As phylogenetic data from various disciplines are becoming widely available, this paradigm shift should be a criterion for designing next‐generation tree visualization tools. This idea should be extended to other fields of biomedical research to develop modern visualization tools that emphasize accessible, interoperable, and reusable scientific data.

## METHODS

The *ggtree* package was developed based on the grammar of graphics implemented in *ggplot2*. Users familiar with *ggplot2* will find *ggtree* very easy to use with little to no learning cost. The experience of using *ggplot2* and *ggtree* is the same and can promote each other. The *ggtree* object defined in the *ggtree* package inherits the *ggplot* object defined in the *ggplot2* package. A *ggtree* object can be constructed using the *ggtree* function, which supports multiple tree‐like classes defined in R [6], such as *phylo*, *phylo4*, *pvclust*, *hclust*, *diana*, *phylog*, *phyloseq*, and so forth. Tree‐associated data can be added to a *ggtree* object with the *%<+%* operator, which links the data to the tree structure and stores the information in the output *ggtree* object. This further expands the integration between the tree‐like classes and related data, facilitating data exploration and presentation, since many tree classes defined for different disciplines in the R language do not support storing external data. From this perspective, a *ggtree* object can be a link that integrates other tree objects with external data and then converts it into a *treedata* object. This makes *tidytree*, *treeio*, *ggtree*, and *ggtreeExtra* [7] more widely applicable as they work with *treedata* objects and can deal with complex phylogenetic annotation by combining different layers of simple annotation, helping to present more diverse tree‐related data. The *%<%* operator extracts visualization directives, which can be applied to the visualization of other tree objects like Word Format Painter. This is an important part of reproducible visualization, yet it has been neglected for a long time. If the visualization styles of graphs published in scientific journals can support such applications, it will greatly promote the presentation of data and beautification of academic graphs. And the *%<%* operator implemented by *ggtree* proves the possibility of this idea.

## AUTHOR CONTRIBUTIONS

Guangchuang Yu conceived the idea. Guangchuang Yu, Shuangbin Xu, Lin Li, and Xiao Luo developed the data structure and function in the package. Shuangbin Xu and Guangchuang Yu wrote the manuscript. Meijun Chen, Wenli Tang, Li Zhan, Zehan Dai, Tommy T. Lam, and Yi Guan contributed to the development. All authors contributed to the final version of the manuscript.

## CONFLICT OF INTEREST

The authors declare no conflict of interest.

## Data Availability

*ggtree* is freely available at https://www.bioconductor.org/packages/ggtree. A complete reference of *ggtree* is available in the online book, https://yulab-smu.top/treedata-book/ [8]. Supplementary materials (figures, tables, scripts, graphical abstract, slides, videos, Chinese translated version and update materials) may be found in the online DOI or iMeta Science http://www.imeta.science/.
